# Essential Role of the Coxsackie - and Adenovirus Receptor (CAR) in Development of the Lymphatic System in Mice

**DOI:** 10.1371/journal.pone.0037523

**Published:** 2012-05-18

**Authors:** Momina Mirza, Mei-Fong Pang, Mohamad Amr Zaini, Paula Haiko, Tuomas Tammela, Kari Alitalo, Lennart Philipson, Jonas Fuxe, Kerstin Sollerbrant

**Affiliations:** 1 Department of Women's and Children's Health, Karolinska Institute and University Hospital, Stockholm, Sweden; 2 Vascular Biology Unit, Department of Medical Biochemistry and Biophysics, Karolinska Institute, Stockholm, Sweden; 3 Molecular/Cancer Biology Laboratory, University of Helsinki, Helsinki, Finland; 4 Department of Cell and Molecular Biology, Karolinska Institute, Stockholm, Sweden; Cincinnati Children's Hospital Medical Center, United States of America

## Abstract

The coxsackie- and adenovirus receptor (CAR) is a cell adhesion molecule predominantly associated with epithelial tight junctions in adult tissues. CAR is also expressed in cardiomyocytes and essential for heart development up to embryonic day 11.5, but not thereafter. CAR is not expressed in vascular endothelial cells but was recently detected in neonatal lymphatic vessels, suggesting that CAR could play a role in the development of the lymphatic system. To address this, we generated mice carrying a conditional deletion of the CAR gene (*Cxadr*) and knocked out CAR in the mouse embryo at different time points during post-cardiac development. Deletion of *Cxadr* from E12.5, but not from E13.5, resulted in subcutaneous edema, hemorrhage and embryonic death. Subcutaneous lymphatic vessels were dilated and structurally abnormal with gaps and holes present at lymphatic endothelial cell-cell junctions. Furthermore, lymphatic vessels were filled with erythrocytes showing a defect in the separation between the blood and lymphatic systems. Regionally, erythrocytes leaked out into the interstitium from leaky lymphatic vessels explaining the hemorrhage detected in CAR-deficient mouse embryos. The results show that CAR plays an essential role in development of the lymphatic vasculature in the mouse embryo by promoting appropriate formation of lymphatic endothelial cell-cell junctions.

## Introduction

The coxsackie- and adenovirus receptor (CAR) was originally identified as a cellular receptor for coxsackie B viruses and type C adenoviruses [1], [2]. Structurally, CAR is related to the Cortical Thymocyte marker in Xenopus (CTX) proteins of the large immunoglobulin family, which also includes the junction adhesion molecules (JAMs) [3], endothelial cell specific adhesion molecule (ESAM) [4] and CAR-like membrane protein (CLMP) [5]. CAR is highly expressed during development in the brain and in the heart, but is rapidly downregulated after birth and thereafter predominantly expressed in tight junctions of epithelial cells [6].

In the heart, CAR localizes to intercalated discs in cardiomyocytes where it plays an essential role as CAR-deficient mouse embryos die around E11.5-E13.5 due to cardiomyocyte dysfunction and heart failure [7], [8]. Importantly, heart-specific deletion of CAR after E11 does not result in a lethal phenotype indicating that CAR is important for heart development only within a short time frame and that CAR may be essential for the development of other organ systems as well [9]. Recent data show that CAR is expressed in human neonatal foreskin lymphatic endothelial cells, where it localizes to cell-cell junctions [10]. *In vitro* studies showed that CAR mediates adhesion between lymphatic endothelial cells and also the capacity of these cells to migrate and form tubes. However, it is not known whether CAR plays a role in the development of the lymphatic system *in vivo*.

The lymphatic system maintains tissue homeostasis by draining fluid from peripheral tissues to the circulation [11]. It is also important for the absorption of lipids from the gastrointestinal tract and for immune cell trafficking during inflammation. Lymphatic dysfunction may lead to lymph edema, impaired immunity and accumulation of subcutaneous fat. The lymphatic system is also the primary route for tumor cell dissemination and therefore plays an important role in cancer metastasis [12].

Development of the lymphatic vasculature begins at embryonic day (E) 9.5–10.5 in mice, after the establishment of a functional blood vasculature [13]. Distinct populations of cardinal vein endothelial cells positive for vascular endothelial growth factor receptor 3 (VEGFR-3), the lymphatic vessel hyaluronan receptor-1 (LYVE-1), and the transcription factors SOX18 and PROX1, commit to the lymphatic lineage and sprout to form lymph sacs in response to VEGF-C. Lymphatic endothelial cells begin to express podoplanin, which promotes platelet aggregation and disconnection of lymphatic vessels from veins during embryonic development [14]. Lymphatic vessels are formed by sprouting from the lymph sacs and are subsequently remodeled into a lymphatic vascular network consisting of blind-ended lymphatic capillaries with specialized button-type junctions, supporting influx of fluid and cells [15], [16]. Collecting lymphatic vessels also acquire luminal valves supporting transport of lymph without backflow [13], [17]. EphrinB2, angiopoietin-2 (Ang-2) and the transcription factor FOXC2 are important for remodeling of the lymphatic vasculature [18], [19], [20]. FOXC2 cooperates with the nuclear factor of activated T cells (NFATc)-1 downstream of VEGFR-3 to control the expression of a set of genes required for the differentiation of lymphatic vessels but is downregulated during maturation of collecting lymphatic vessels, leading to decreased expression of PROX1, VEGFR-3 and LYVE-1 at later stages [21]. The lymphatic vasculature develops and matures during the postnatal period and in adult tissues, growth of lymphatic vessels is normally restricted to pathological conditions characterized by tissue remodeling, such as cancer and chronic inflammation.

We wanted to elucidate whether CAR could play a role in development of the lymphatic system in the mouse. To study this we generated tamoxifen-inducible CAR deficient mice, which allowed us to delete *Cxadr* at different time points after E11, when CAR expression in cardiomyocytes was not essential for heart development. The results demonstrate an essential role of CAR for the development of the lymphatic vasculature.

## Results

### CAR-deficiency during a critical time window of mouse development leads to subcutaneous edema and hemorrhage

**Figure 1:**
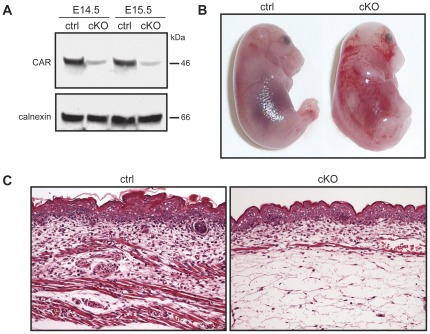
CAR deficiency in mouse embryos after heart development causes subcutaneous edema, hemorrhage and lethality. (A) Western blot analysis of CAR protein levels in E14.5 and E15.5 embryos from *F/F;Cre* (cKO, n = 3) and littermate *F/F* control (ctrl, n = 3) animals from two litters after administration of tamoxifen at E12.5. Primary CAR antibody was RP 291. An antibody against calnexin was used as a loading control. (B) Macroscopic images of E16.5 embryos given tamoxifen at E12.5 showing subcutaneous edema and hemorrhage in cKO (n = 12) embryos but not in littermate *F/F* (n = 18) control embryos (ctrl), which appeared normal. Embryos were from 5 litters. Images were taken with a 4× objective. (C) Histological analysis of transverse sections of E16.5 embryos given tamoxifen at E12.5 showing subcutaneous edema in tamoxifen-treated cKO (n = 3) embryos while the littermate *F/F* controls (ctrl, n = 3) were normal. Embryos were from 3 litters. Images were taken with a 20× objective. Scale bar = 50 µm.

Mice carrying floxed *Cxadr* alleles (*CxadrFlox/Flox*) (*F/F*) were generated, as recently described [22]. To induce recombination between the loxP sites in mouse embryos, *F/F* females were crossed with *F/F;Cre* males. Additional breeding created the mouse line *F/F;Cre* that was backcrossed three times onto C57Bl/6J and then used for experiments. Starting at E12.5, pregnant females were given tamoxifen by intraperitoneal injections on two consecutive days. Animals were sacrificed at different time-points from E14.5 to E18.5. Genotyping of genomic DNA isolated from the tail demonstrated an equal distribution of *F/F* and *F/F;Cre* embryos, as expected, and an efficient Cre-mediated recombination in *F/F;Cre* embryos (conditional knockout, cKO), with none in *F/F* littermate controls (ctrl) [22]. Western blotting analysis of protein extracts from decapitated embryos demonstrated significantly reduced CAR levels in cKO embryos compared to ctrl embryos at E14.5 and E15.5 (Fig. 1A). Importantly, CAR protein levels in tamoxifen-treated *F/F* and *+/+;Cre* (mice carrying wild type *Cxadr* alleles) control embryos were indistinguishable from wildtype embryos demonstrating that neither tamoxifen treatment itself nor the presence of the Cre protein without loxP recombination sites could disrupt expression from *Cxadr* [22].

Gross differences between CAR cKO embryos compared to control embryos could be seen from E16.5 (Fig. 1B and Table 1). Both male and female cKO embryos were smaller than their littermate controls and displayed macroscopic hemorrhage and subcutaneous edema. Table 1 shows the proportion of embryos with visible, phenotypic changes. Genotyping of the two litters examined at E18.5 demonstrated that none of the living embryos were cKO embryos. At this timepoint most cKO embryos were already in advanced states of resorption, which made analysis of edema and hemorrhage very difficult. In Table 1 we therefore choosed to exclude these embryos and include only embryos with clear visible, phenotypic changes. Microscopic inspection of the limbs indicated that most cKO embryos did not develop beyond E17.5 (data not shown). Histological analyses revealed subcutaneous edema in cKO embryos starting from E15.5 (Fig. 1C). No signs of cardiac dilation or structural abnormalities in the heart similar to those described in hearts of CAR^−/−^ mice before E11.5 [8] [7], or abnormalities in any other tissues, were observed. No abnormal phenotype was observed in tamoxifen-treated heterozygous *F/+;Cre* animals or in *+/+;Cre* controls (data not shown).

**Table 1:** The proportion of embryos with macroscopic phenotypic changes following tamoxifen administration at E12.5.

Embryonic day (E)	ctrl		cKO	
	n	%	n	%
E16.5 am	0 (10)	0	0 (12)	0
E16.5 pm	0 (18)	0	8 (20)	40
E17.5	0 (11)	0	10 (17)	59
E18.5	0 (7)	0	2 (2)	100

Embryonic day (E) indicate day of analysis. The morning and afternoon of E16.5 is indicated as am and pm, respectively. Ctrl and cKO are tamoxifen-treated embryos with genotypes F/F and F/F;Cre, respectively. Animals with macroscopic phenotypic changes are indicated as number of animals (n) as well as % of animals. The total number of embryos analyzed is indicated within brackets. Phenotypic changes include edema, hemorrhage and reduced crown-rump length.

Administration of tamoxifen to *F/F;Cre* mice from E13.5 and onwards did not result in edema formation or hemorrhage, and was not lethal (data not shown). Together with previous data showing that heart-specific deletion of CAR after E11 is not lethal, our results indicated that CAR plays an essential role for the formation of other parts of the cardiovascular system during a critical time period of development.

### CAR is expressed in lymphatic but not vascular endothelial cells during mouse development

To investigate this further we performed immunofluorescent staining of whole embryos, or whole-mount skin preparations, to study the expression of CAR in subcutaneous blood and lymphatic vessels of embryos at different stages of development. Blood and lymphatic vessels were visualized and distinguished from each other through their well-established expression of CD31, which is high in blood vessels (CD31^high^) and low in lymphatic vessels (CD31^low^), and LYVE-1, which is expressed in lymphatic vessels (LYVE-1^pos^) but not in blood vessels (LYVE-1^neg^).

**Figure 2:**
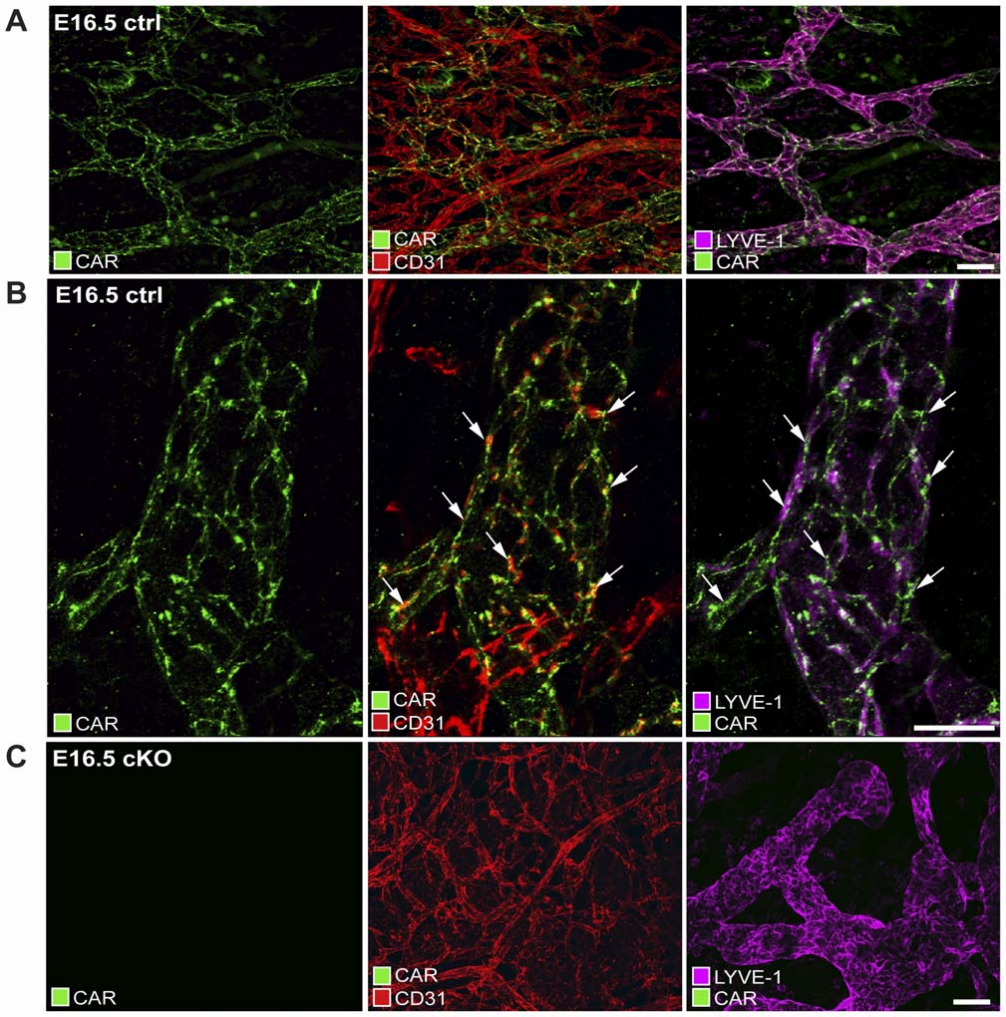
CAR is expressed in lymphatic endothelial cells during mouse development and localizes to cell-cell junctions. Confocal images of whole-mount skin preparations from wildtype (A, B) (n = 3) and cKO (C) (n = 3) E16.5 embryos stained by immunofluorescence with the antibodies rabbit anti-CAR IG1 (green), hamster anti CD31 (red) and rat anti LYVE-1 (purple) to visualize CAR expression in blood vessels (CD31highLYVE-1neg) and lymphatic vessels (CD31lowLYVE-1pos). Animals were from 3 different litters (A) Low magnification images showing CAR expression in lymphatic vessels but not in blood vessels. Scale bar = 50 µm (B). High-magnification images showing distribution and partial co-localization of CAR with CD31, and LYVE-1 at lymphatic endothelial cell-cell junctions (arrows). Scale bar = 50 µm. (C) Low magnification images showing efficient downregulation of CAR in lymphatic vessels of cKO embryos. Scale bar = 50 µm.

CD31^high^/LYVE-1^neg^ blood vessels were present in the skin at all time points between E13.5 and E16.5 (Fig. S1). In comparison, CD31^low^/LYVE-1^pos^ lymphatic vessels were almost absent in the skin of E13.5 control embryos but were present from E14.5 and onwards, demonstrating that the lymphatic vasculature of the skin was being formed during this time window. No expression of CAR was detected in blood vessels at any of the time points (Figs. 2A, and S2). In contrast, positive CAR staining was detected in subcutaneous lymphatic vessels at E.14.5, E.15.5, and E.16.5 (Figs. 2A, and S2). Similar to the distribution in epithelial cells, CAR localized to cell-cell junctions in lymphatic endothelial cells, where it partially co-localized with CD31 and LYVE-1 (Fig. 2B). No CAR staining was found in the skin of E13.5 control embryos (Fig. S2). In the skin of adult mice, CAR expression was readily detected in the epithelium, but absent in the lymphatic vasculature (Fig. S2). These results are in agreement with our previous studies showing lack of CAR expression in lymphatic vessels of adult mice [23].

The results demonstrated that CAR is expressed at lymphatic endothelial cell junctions during the initial phase of lymphatic vessel development. However, as the lymphatic system is remodeled into a hierarchical network of lymphatic vessels, CAR expression declines to undetectable levels. Thus, the formation of the lymphatic system and the expression of CAR in lymphatic endothelial cells coincided with a period of mouse development between E.12.5–E16-5, when mouse embryos were sensitive to CAR deficiency. Together, this suggested that the subcutaneous edema observed in the cKO embryos was due to impaired tissue homeostasis caused by non-existing or dysfunctional lymphatic vessels.

### Deformed and dilated lymphatic vessels in CAR-deficient mouse embryos

Immunohistochemical staining for LYVE-1 in embryo sections revealed no major differences in the distribution of lymphatic vessels in cKO versus control embryos at E16.5 (data not shown). Thus, lymphatic vessels were not absent in CAR-deficient embryos. Imaging of whole-mount skin preparations stained by immunofluorescence and analyzed by high-resolution confocal microscopy revealed no detectable expression of CAR in lymphatic vessels of the CAR cKO embryos, thus verifying that tamoxifen-mediated deletion of the CAR gene was efficient in lymphatic endothelial cells (Fig. 2C).

**Figure 3:**
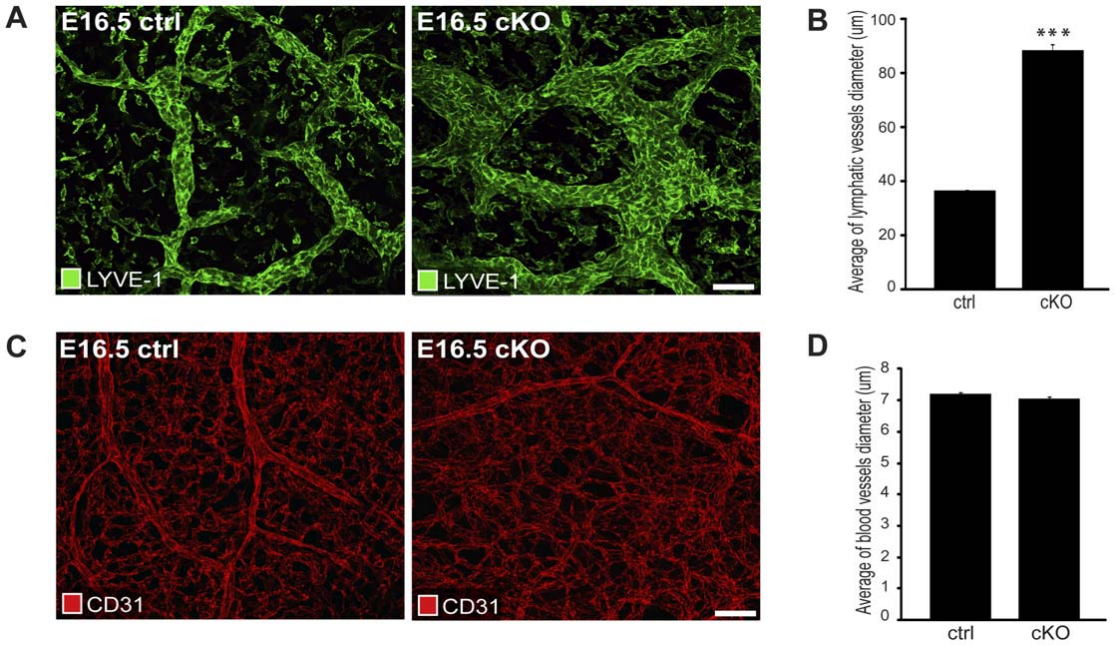
Lymphatic vessels of CAR deficient mouse embryos are structurally abnormal. (A, C) Confocal immunofluorescence images of whole-mount skin preparations from cKO (n = 5) and littermate *F/F* controls (ctrl, n = 4) at E16.5 co-stained with the antibodies rabbit anti-LYVE-1 (green) and hamster anti-CD31 (red) to visualize lymphatic vessels (A) and blood vessels (C), respectively. Embryos were from 2 litters. Scale bar = 50 µm. (B, D) Measure of vessel diameter. Data is presented as means ± SEM with at least four to six mice per group. (A) Immunofluorescence images showing dilated lymphatic vessels in cKO compared to ctrl embryos. (B) Bar graph showing significantly increased average diameter of subcutaneous lymphatic vessels in cKO versus *F/F* control (ctrl) embryos at E16.5. P<0.001. (C) Immunofluorescence images showing no differences in blood vessels in cKO compared to ctrl embryos. (D) Bar graph showing no significant differences in average diameter of subcutaneous blood vessels in cKO versus *F/F* control (ctrl) embryos at E16.5. P = 0.76.

Lymphatic vessels in the skin of cKO embryos were structurally abnormal and dilated compared to lymphatic vessels in control embryos (Fig. 3A and Fig. 2C (compare to Fig. 2A)). Quantification of vessel size revealed that the average lymphatic vessel diameter was more than doubled in cKO embryos compared to littermate controls (Fig. 3B). Analyses performed at E14.5 and E15.5 showed similar defects in cKO embryos thus demonstrating that lymph vessel defects preceded the observed subcutaneous edema (Fig. S3). Blood vessels analyzed at the same embryonic age appeared normal and were not enlarged (Fig. 3C,D). These findings are consistent with those from a previous study, in which CAR-deficiency was shown to affect lymphatic tube formation *in vitro* [10].

### Impaired formation of lymphatic endothelial cell-cell junctions in CAR-deficient mice

**Figure 4:**
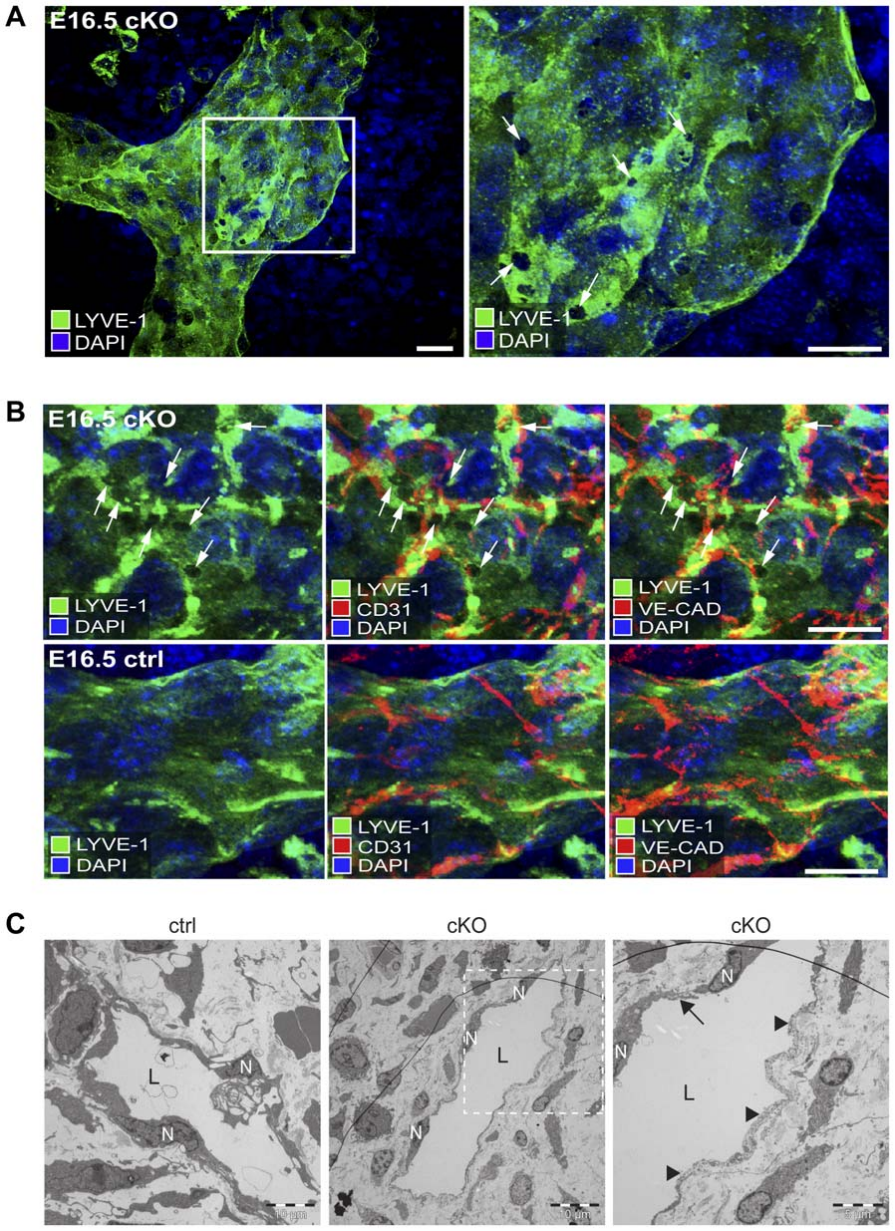
Lymphatic vessels of CAR deficient mouse embryos display endothelial gaps. (A, B) High-resolution confocal images of whole-mount skin preparations from E16.5 cKO (n = 3) and littermate *F/F* control (ctrl, n = 3) embryos stained in three independent experiments by immunofluorescence with antibodies to visualize lymphatic vessels (LYVE-1, green) and junctions between lymphatic endothelial cells (CD31/VE-cadherin, red). DAPI was used to visualize nuclei (blue). (A) Immunofluorescence image showing a dilated LYVE-I positive lymphatic vessel in an E16.5 cKO embryo (left). Gaps between endothelial cells are present and even more visible at higher magnification (arrows in right image, which is a magnification of the white box outlined in the left image). Primary antibody was rat anti-LYVE-1. Scale bar = 50 µm. (B) Top panel: High-magnification images of a dilated LYVE-I positive lymphatic vessel from an E16.5 cKO embryo showing the presence of lymphatic endothelial gaps (arrows) at, or close to, cell-cell junctions visualized by co-staining with antibodies against CD31 (red, middle image) or VE-cadherin (red, right image). Bottom panel: no gaps were present in similarly stained *F/F* littermate controls (ctrl). Primary antibodies were rabbit anti-LYVE-1, hamster anti CD31 and rat anti VE-cadherin. Scale bar = 150 µm. (C) Transmission electron microscopy (TEM) images of subcutaneous lymphatic vessels (L) in E16.5 cKO (n = 4) and littermate *F/F* control (ctrl, n = 4) embryos. Embryos were from two litters. In ctrl embryos, lymphatic endothelial cells with clearly visible nuclei (N) formed an intact lining around the entire lumen of the lymphatic vessels. In cKO embryos, lymphatic endothelial cell nuclei were smaller and the cells appeared locally damaged (arrow) and did not form an intact lining of the vessel lumen. Regions of the luminal surface were devoid of lymphatic endothelial cells (arrowheads). Scale bars = 10 µm (left and middle images), 5 µm (right image).

**Figure 5:**
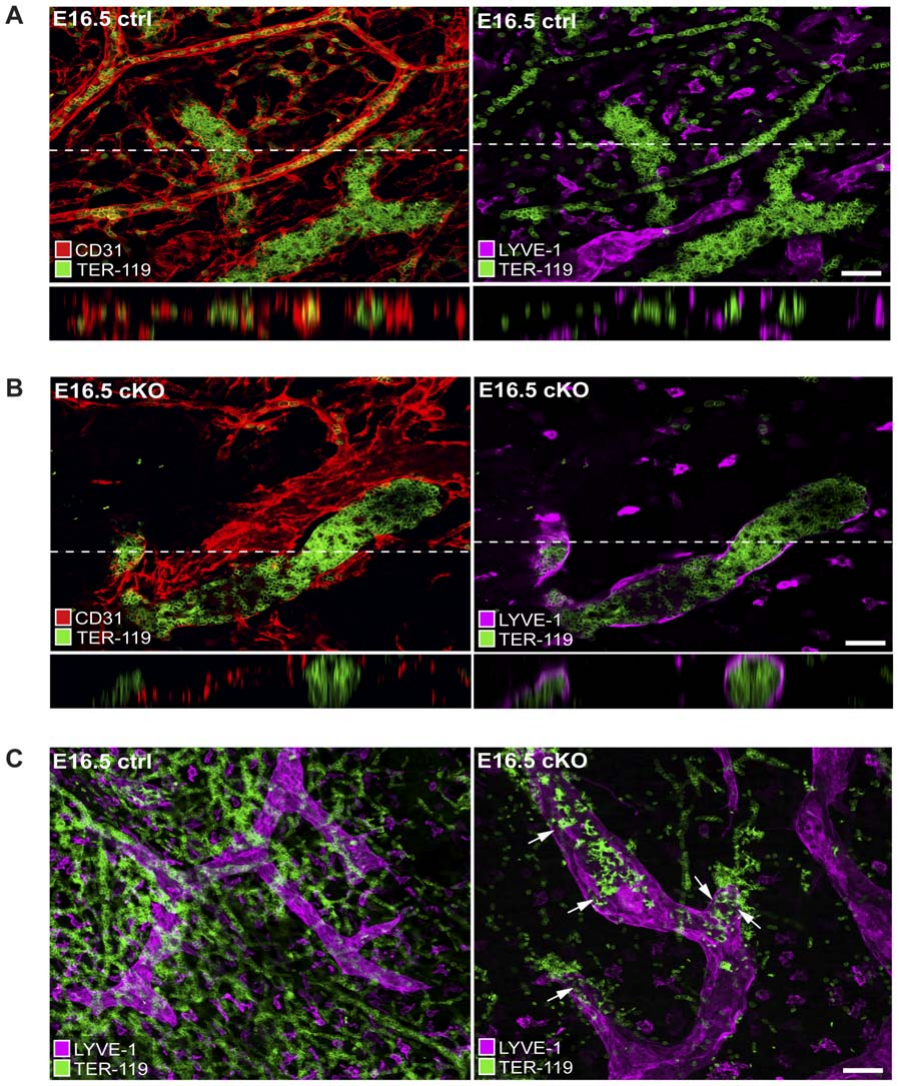
CAR deficiency during mouse development causes incomplete separation of the blood and lymphatic systems. (A–C) Confocal images of whole-mount skin preparations from E16.5 cKO (n = 3) and littermate *F/F* control (ctrl, n = 3) embryos stained in three independent experiments by immunofluorescence with rat anti-TER 119 (TER, green) to visualize erythrocytes, and the vascular markers hamster anti-CD31 (red) and rabbit anti-LYVE-1 (purple) to visualize blood vessels (CD31highLYVE-1neg) and lymphatic vessels (CD31lowLYVE-1pos). Scale bar = 50 µm. (A, B) Images showing staining for TER in blood but not in lymphatic vessels in ctrl embryos (A), and staining for TER in dilated lymphatic vessels in cKO embryos (B). Lower panels show z projections of the upper images at levels indicated by the white lines. (C) Images showing leakage of TER-positive erythrocytes from dilated lymphatic vessels in cKO but not in ctrl embryos.

More detailed analysis of LYVE-1 stained whole-mount skin preparations from E16.5 cKO embryos revealed the presence of gaps and holes at or close to junctions between endothelial cells of subcutaneous lymphatic vessels (Fig. 4A, B). Such gaps were not identified in lymphatic vessels from control embryos (Fig. S4). In some areas, larger wholes were found in lymphatic vessels from CAR cKO embryos (Fig. 5C). These results indicated that CAR-deficiency resulted in impaired cell-cell adhesion and formation of junctions between lymphatic endothelial cells.

To further analyze the integrity of lymphatic vessels in cKO embryos at the ultrastructural level, skin tissues from cKO and control embryos at E16.5 were analyzed by transmission electron microscopy. In control embryos, lymphatic endothelial cells were connected to each other forming an intact lining around the entire lumen of the lymphatic vessels (Fig. 4C). In cKO embryos, lymphatic vessels appeared damaged and necrotic endothelial cells with small nuclei were frequently observed. Regions of the luminal surface of lymphatic vessels were devoid of lymphatic endothelial cells.

Together, these results indicated that CAR is important for the formation of junctions between lymphatic endothelial cells. Since no macroscopic edema or defects in lymphatic development were observed when CAR knockdown was induced at later time points (from E13.5), and since CAR expression was high in developing lymphatic vessels but low in maturating vessels we conclude that CAR plays an essential role for the formation of the lymphatic system during a critical developmental period when the lymphatic system is being formed.

### Impaired separation between blood and lymphatic vessels in CAR-deficient mouse embryos

While leaky lymphatic vessels could explain the subcutaneous edema observed in the cKO embryos the mechanism involved in causing hemorrhage remained unclear. A critical part of the formation of the lymphatic system is the separation between the blood and the lymphatic vasculature to create two separate circulation systems. To investigate whether incomplete separation between blood and lymphatic vessels contributed to the phenotype of cKO embryos we stained whole-mount skin preparations of E16.5 embryos with the erythrocyte marker TER-119. As expected, ubiquitous TER-119 staining was seen in blood vessels but not in lymphatic vessels in control animals (Fig. 5A). However, in the cKO embryos, TER-119 staining was frequently observed in lymphatic vessels demonstrating the presence of erythrocytes in the lymphatic system (Fig. 5B). Blood-filled lymphatic vessels were often found in close proximity to blood vessels. A close inspection of blood-filled lymphatic vessels revealed interstitial leakage of erythrocytes at regions of lymphatic vessels where gaps were present, thus forming regions of hemorrhage (Fig. 5C).

These results showed that CAR expression at lymphatic endothelial cell-cell junctions is essential for proper formation and function of lymphatic vessels during development and also, for complete separation between the blood and the lymphatic vasculature.

## Discussion

The aim of this study was to analyze the role of CAR in embryo development after E11.5. For this purpose we constructed a conditional knockout (cKO) mouse strain, in which the CAR gene was deleted in all embryonic tissues following intraperitoneal administration of tamoxifen to pregnant females. We found that deletion of the CAR gene from E12.5, at a time point when CAR is no longer essential for cardiac development [9], caused subcutaneous edema, hemorrhage, and embryonic death. This suggested that CAR was important for the development of additional parts of the cardiovascular system.

Similar to previous reports, CAR expression was not found in blood vessels at any time point of development. Subcutaneous blood vessels appeared structurally normal in CAR-deficient embryos displaying subcutaneous edema and hemorrhage. Together, these results suggested that CAR-deficiency did not primarily affect blood vessels. In contrast, the observed subcutaneous edema and hemorrhage in CAR-deficient mouse embryos were associated with the presence of structurally and functionally abnormal lymphatic vessels.

Lymphatic vessels in CAR-deficient embryos were dilated and contained gaps and holes at junctions between lymphatic endothelial cells. Thus, we conclude that CAR plays an important role for proper formation of cell-cell junctions between lymphatic endothelial cells at a time point when lymphatic vessels are formed. This is supported by recent *in vitro* data showing that siRNA-mediated knockdown of CAR in cultured lymphatic endothelial cells results in impaired formation of intercellular junctions and increased permeability across the endothelial cell layer [10]. The results showing the presence of larger wholes with necrotic lymphatic endothelial cells in areas of lymphatic vessels from the CAR cKO embryos could reflect that improper formation of tight junctions may lead to degeneration and loss of lymphatic endothelial cells. Interestingly, knockdown of CAR from E13.5 was not embryonic lethal. In addition, CAR expression was not detected in lymphatic endothelial cells in adult mice. Thus, CAR seems to play an essential role for lymphatic development during a developmental period when junctions between lymphatic endothelial cells are formed. However, as interactions between lymphatic endothelial cells are stabilized during the formation a fully matured lymphatic network, CAR is no longer needed. It will be of interest for the future to analyze whether CAR is upregulated and important for the formation of new lymphatic vessels during pathological lymphangiogenesis, which frequently is seen both in tumors and in conditions of chronic inflammation.

The hemorrhage observed in CAR deficient mouse embryos was initially difficult to explain since blood vessels appeared normal. However, as we discovered blood circulating inside and leaking out from defective lymphatic vessels we concluded this to be a likely mechanistic explanation for the hemorrhage. The more precise mechanism by which CAR mediates separation between the blood and lymphatic systems remains to be determined. It could be that CAR-mediated formation of junctions between lymphatic endothelial cells is important for complete closure between the systems. On the other hand, it could also relate to a yet undiscovered role of CAR. Similar to our results, incomplete separation of the vascular and lymphatic systems was previously reported in mice disrupted of *Syk*, *SLP-76*, *kindlin-3* or *podoplanin*, genes required for the function of platelets [13], [14]. Among proteins encoded by these genes, podoplanin is also, similar to CAR, a transmembrane protein expressed in lymphatic, but not vascular endothelial cells. Interaction between lymphatic endothelial podoplanin and circulating platelets was shown to activate and aggregate platelets leading to closure of the junction between the cardinal vein and the developing lymph sacs [14]. Recently, it was reported that CAR is expressed on human platelets and involved in mediating adenovirus attachment to these cells [24]. It is therefore tempting to speculate that CAR, similar to podoplanin, is involved in interactions between lymphatic endothelial cells and platelets and that failure of such interactions to form represent a mechanism of the incomplete separation between the blood and lymphatic systems in cKO embryos. However, to completely elucidate this would require cell-type specific deletion or rescue of CAR in lymphatic endothelial cells and platelets.

In summary, this study demonstrates an essential and previously undiscovered role for CAR in the development of the lymphatic system in the mouse. CAR deficiency during a critical time window between E12.5–E15.5 leads to formation of structurally abnormal and functionally impaired lymphatic vessels. The mechanism involves incomplete formation of lymphatic endothelial cell-cell junctions and as a result, failure of these cells to interact with each other. In addition, CAR is required for proper separation of the blood and lymphatic vasculature, and CAR deficiency during this critical time window leads to ectopic blood flow in the lymphatic system, edema and hemorrhage due to leaky lymphatic vessels. Based on our data, recent results showing that CAR is expressed in platelets [24], and the discovery that platelets play a critical role in the separation between the blood and lymphatic vasculature (reviewed in [25]), it is possible that this separation process requires CAR-mediated interactions between lymphatic endothelial cells and platelets.

Future studies should give further insights into the molecular and cellular processes involved, as well as possible links to human disease.

## Materials and Methods

### Ethics Statement

All animal experiments were approved by the animal ethical committee and performed in accordance with its regulations.

### Generation of CAR deficient mice and genotyping

Floxed mice (*F/F*) harboring two loxP sites flanking *Cxadr* exon-2 were generated at the MCI/ICS (Mouse Clinical Institute - Institut Clinique de la Souris) in France; as described [22]. *F/F* mice were then crossed with the transgenic mouse line B6.Cg-Tg (CreEsr1)5 AmC/J (purchased from The Jackson Laboratory, stock number 004682) expressing a tamoxifen-inducible Cre-ERTM fusion protein under the control of a chicken ß actin/cytomegalovirus (CMV) promoter [26]. Additional breeding created the mouse line *F/F;Cre* that was backcrossed three times onto C57Bl/6J and then used for experiments. PCR was used for genotyping as described in [22].

### Nomenclature

The following nomenclature is used for mice throughout the paper. *+/+* animals are wt C57Bl/6J mice, cKO are tamoxifen-treated animals with the genotype *F/F;Cre*, ctrl are control animals with genotype *+/+* (wildtype) or *F/F* as indicated in the text, *+/+;Cre* animals express the Cre recombinase but do not contain any loxP sites. CAR and *Cxadr* is used throughout the paper and refers to the protein and the *Cxadr* gene, respectively.

### Animal procedures

Embryos older than E11 were sacrificed by decapitation due to ethical considerations. The embryonic age was determined such that the day of the vaginal plug was E 0.5. Tamoxifen (Sigma T-5648) was dissolved in corn oil (Sigma C-8267) to a final concentration of 10 mg/ml by vigorous shaking at 45°C for 2 hours. Tamoxifen was prepared fresh every week. Timed plugged females were injected intraperitoneally with 2 mg tamoxifen/40 g mice for two consecutive days at E12.5 and E13.5.

### Antibodies

Primary antibodies used for immunofluorescence staining were; CAR: rabbit anti-CAR RP 291 [23], rabbit anti-CAR IG1 [23]; blood and/or lymphatic vessels: hamster anti–CD31 (clone 2H8; Chemicon), rabbit anti-LYVE-1 (Abcam), rat anti-LYVE-1 (Novus Biologicals); adherens junctions: rat anti-VE-cadherin (clone BV13, a kind gift to JF from E. Dejana, FIRC Institute, Milan, Italy); erythrocytes: rat anti-TER119 (eBioscience). Wholemount tissues were mounted in Vectashield mounting media with DAPI (Vectalabs). Primary antibodies used for Western analyses were rabbit anti-CAR RP 291 [23] and rabbit anti-calnexin [27]. FITC, Cy3, or Cy5-labelled secondary antibodies for immunofluorescence were from Jackson ImmunoResearch Laboratories, and secondary antibody for Western analysis was HRP-labeled donkey anti-rabbit Ig (NA934V, GE healthcare, UK).

### Western blotting

Whole embryo without the head was homogenized in lysis buffer (50 mmol/L Tris-HCl (pH 7.5), 137 mmol/L NaCl, 0.5% Triton X-100, and EDTA-free 1× complete protease inhibitor from Roche Molecular Biochemicals) on ice, sonicated, and incubated on ice for 20 min before centrifugation at maximum speed in an Eppendorf centrifuge (Hamburg, Germany) at +4°C for 15 min. The lysate was removed and run on a 10% SDS-PAGE gel under reducing conditions, and transferred to PROTRAN nitrocellulose transfer membrane (Schleicher & Schuell, Dassel, Germany). Membranes were incubated in blocking buffer (1× PBS with 0.1% Tween 20 and 5% dry milk) at room temperature and then incubated with primary and secondary antibody diluted in blocking buffer. Peroxidase activity was detected using enhanced chemiluminescence and Hyperfilm ECL (Amersham Biosciences).

### Whole mount imaging and immunofluorescence analyses

Pregnant mice were sacrificed and embryos were taken out at day E.14.5, E15.5, and E16.5. The dorsal skin of embryos was excised. For adult skin whole-mount, hair at the back of the mice was shaved prior to excision of the dorsal skin. Skin was immersed in 4% paraformaldehyde in PBS (pH 7.4) for 3 hours at room temperature. Skin was washed with PBS and stained with primary and secondary antibodies diluted in incubation buffer (0.3% Triton X-100, 0.2% bovine serum albumin, 5% normal goat serum and 0.1% sodium azide). Specimens were mounted in Vectashield mounting media with DAPI (Vectalabs) and visualized with confocal microscope (LSM-700; Carl Zeiss Microimaging, Inc) using LSM Image Examiner.

### Histological analysis of mouse tissues

Pregnant mice were sacrificed and embryos were dissected and fixed in Bouin's solution (Sigma HT10132) at room temperature overnight. Next day the embryos were washed with 70% ethanol several times and embedded in paraffin, sectioned and stained with hematoxylin and eosin according to standard procedures. Light microscopy was performed on a Nikon Eclipse 800; TEKNO microscope. A pathologist examined tissues and evaluated phenotypic abnormalities in tissues including the heart.

### Measure of vessel diameter

Images of whole mount skin preparations from E16.5 cKO and littermate *F/F* control (ctrl) embryos stained by immunofluorescence with antibodies against CD31 and LYVE-1 were taken using a Zeiss LSM-700 confocal microscope. Blood and lymphatic vessels were identified as CD31high/LYVE-1neg and CD31low/LYVE-1pos, respectively. Images were taken from three random areas of four cKO and six *F/F* control animals and imported into the ImageJ software (http://rsb.info.nih.gov/ij) for measurements. Measurement of the diameter of blood vessels was based on images taken with a 20× objective while measurement of the diameter of lymphatic vessels was based on 10× objective. The average diameter of blood and lymphatic vessels diameters was calculated from the individual measurements.

### Statistics

Data were presented as means ± SEM with at least four to six mice per group. Statistical analyses were determined by using Student's *t* test. P<0.05 considered as significant.

### Transmission electron microscopy (TEM)

Skin tissue was dissected and small pieces were fixed in 2% glutaraldehyde +0.5% paraformaldehyde in 0.1 mol/L sodiumcacodylate buffer containing 0.1 mol/L sucrose and 3 mmol/L CaCl_2_, pH 7.4 at room temperature for 30 min followed by 24 hours at 4°C. Specimens were rinsed in 0.15 mol/L sodiumcacodylate buffer containing 3 mmol/L CaCl_2_, pH 7.4 postfixed in 2% osmium tetroxide in 0.07 mol/L sodiumcacodylate buffer containing 1.5 mmol/L CaCl_2_, pH 7.4 at 4°C for 2 hour, dehydrated in ethanol followed by acetone and embedded in LX-112 (Ladd, Burlington, Vermont, USA). Semithin sections were cut and stained with toludinblue and used for light microscopic analysis. Ultrathin section (approximately 40–50 nm) were cut and contrasted with uranyl acetate followed by lead citrate and examined in a Leo 906 transmission electron microscope at 80 kV. Digital images were taken by using a Morada digital camera (Soft Imaging System, GmbH, Münster, Germany). A pathologist examined tissues and evaluated structural abnormalities of tissues including the heart.

## Supporting Information

Figure S1 **Subcutaneous lymphatic vessels are formed during a time period of mouse development between E13.5 and E16.5.** Confocal images of whole-mount skin preparations from wildtype mouse embryos (n = 4) of ages E13.5, E14.5, E15.5, and E16.5 stained by immunofluorescence with antibodies hamster anti-CD31 (red) and rat anti LYVE-1 (purple) to visualize blood vessels (CD31highLYVE-1neg) and lymphatic vessels (CD31lowLYVE-1pos). Blood vessels were detected at all embryonic ages and gradually increased in numbers from E13.5 to E16.5. Lymphatic vessels were almost completely absent at E13.5, but were detected and increased in numbers from E14.5 and onwards. Scale bar = 50 µm.
(TIFF)

Figure S2 **CAR expression in lymphatic vessels is restricted to a period during development.** Confocal immunofluorescence images of whole-mount skin preparations from wild-type embryos at E13.5, E14.5 and E15.5, and from adult mice. CAR expression (green) was readily detected in lymphatic vessels (purple, CD31lowLYVE-1pos) but not in blood vessels (red, CD31highLYVE-1neg) at E14.5 and E15.5. CAR expression in adult mice was clearly detected in the skin epithelium, but not in lymphatic vessels. Scale bar = 50 µm.
(TIFF)

Figure S3 **Structurally abnormal lymphatic vessels in CAR deficient embryos at E14.5 and E15.5.** High-resolution confocal images of whole-mount skin preparations from CAR cKO and littermate *F/F* controls (ctrl) at E14.5 and E15.5 stained by immunofluorescence for LYVE-1 (green). Abnormal lymphatic vessels are seen in cKO embryos but not in controls at both time points. Scale bar = 50 µm.
(TIFF)

Figure S4 **Lymphatic vessels of E16.5 ctrl embryos have no endothelial gaps.** High-resolution confocal images of whole-mount skin preparations from E16.5 *F/F* control embryos stained by immunofluorescence for LYVE-1 (green). DAPI was used to visualize nuclei (blue). No endothelial gaps were present in lymphatic vessels of ctrl embryos. Scale bar = 150 µm.
(TIFF)
