# Supplementary figures and images for: Essential Role of the Coxsackie - and Adenovirus Receptor (CAR) in Development of the Lymphatic System in Mice
Source: PLoS One. 2012 May 18;7(5):e37523. doi: 10.1371/journal.pone.0037523 (PMC3356332; doi:10.1371/journal.pone.0037523)

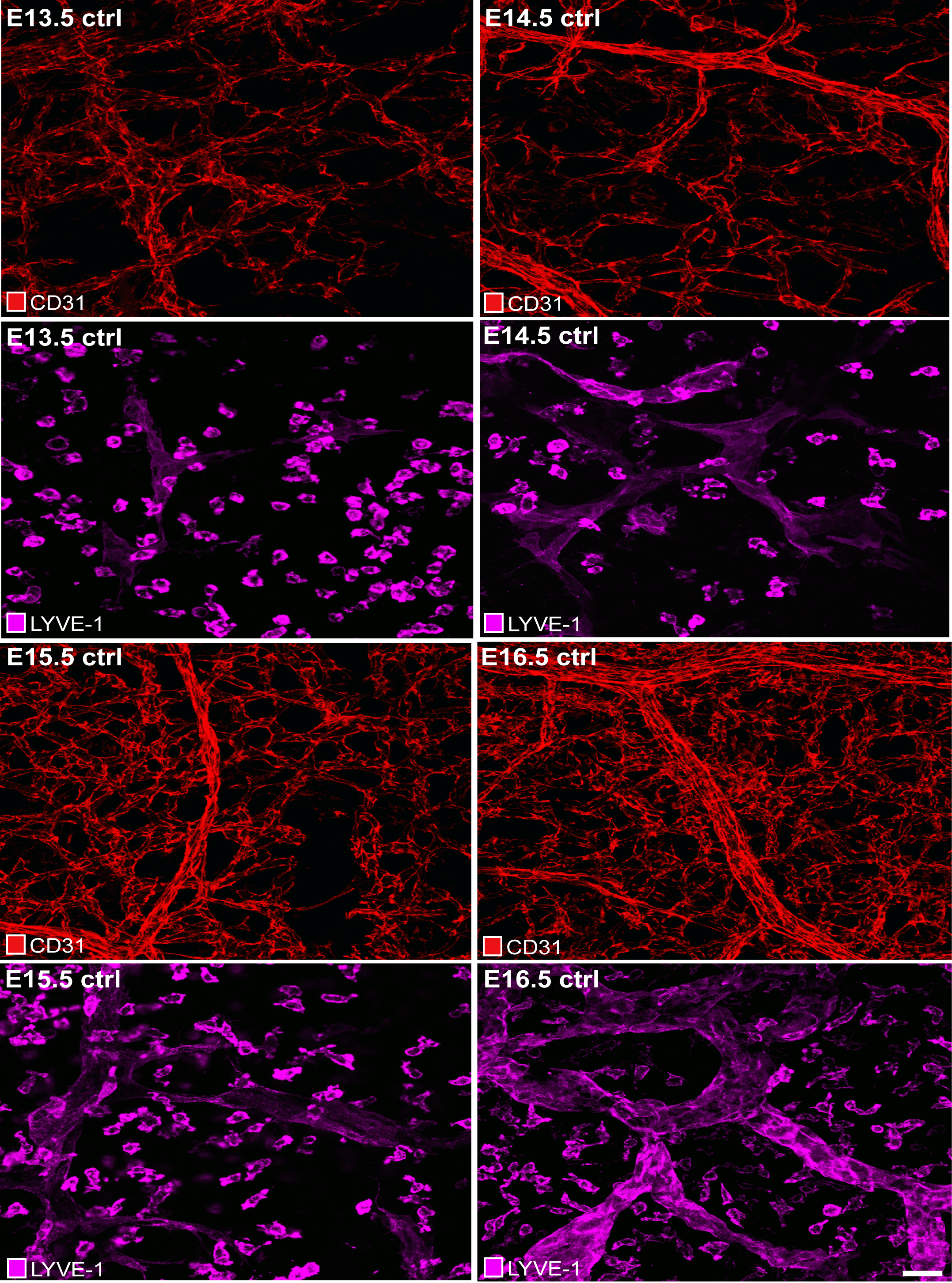

Supplement: Figure S1 — Subcutaneous lymphatic vessels are formed during a time period of mouse development between E13.5 and E16.5. Confocal images of whole-mount skin preparations from wildtype mouse embryos (n = 4) of ages E13.5, E14.5, E15.5, and E16.5 stained by immunofluorescence with antibodies hamster anti-CD31 (red) and rat anti LYVE-1 (purple) to visualize blood vessels (CD31highLYVE-1neg) and lymphatic vessels (CD31lowLYVE-1pos). Blood vessels were detected at all embryonic ages and gradually increased in numbers from E13.5 to E16.5. Lymphatic vessels were almost completely absent at E13.5, but were detected and increased in numbers from E14.5 and onwards. Scale bar = 50 µm. (TIFF) [file pone.0037523.s001.tif]

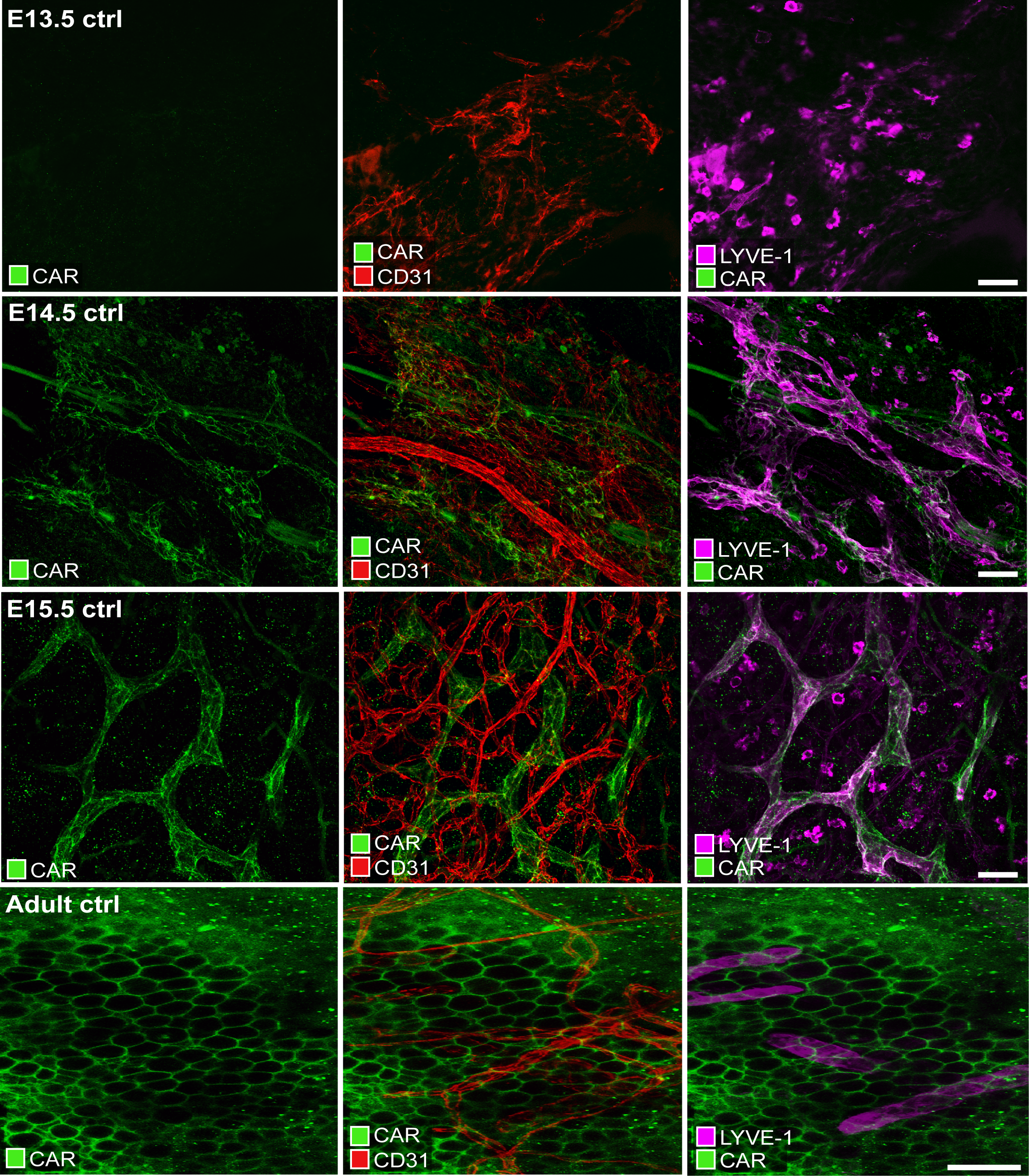

Supplement: Figure S2 — CAR expression in lymphatic vessels is restricted to a period during development. Confocal immunofluorescence images of whole-mount skin preparations from wild-type embryos at E13.5, E14.5 and E15.5, and from adult mice. CAR expression (green) was readily detected in lymphatic vessels (purple, CD31lowLYVE-1pos) but not in blood vessels (red, CD31highLYVE-1neg) at E14.5 and E15.5. CAR expression in adult mice was clearly detected in the skin epithelium, but not in lymphatic vessels. Scale bar = 50 µm. (TIFF) [file pone.0037523.s002.tif]

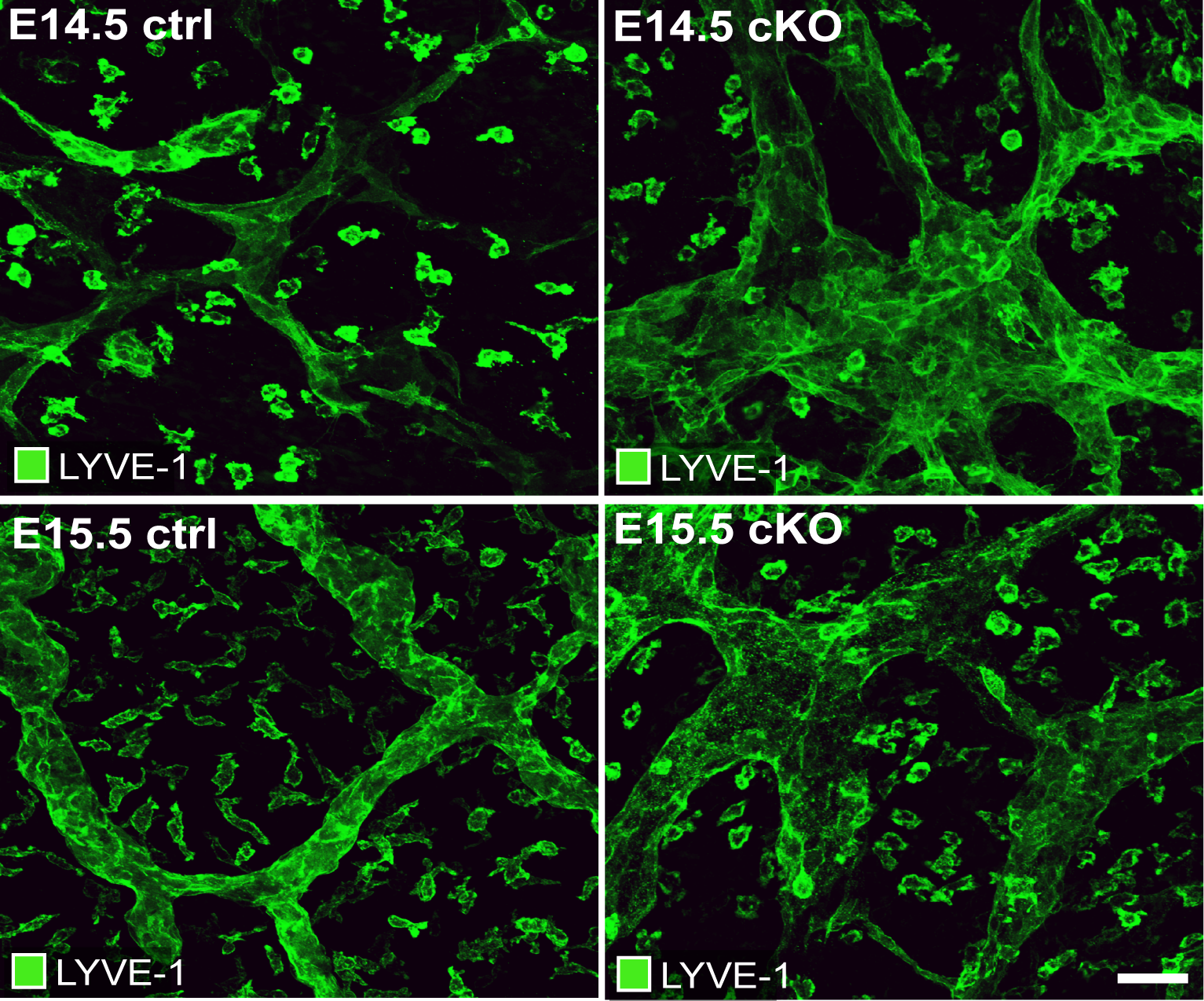

Supplement: Figure S3 — Structurally abnormal lymphatic vessels in CAR deficient embryos at E14.5 and E15.5. High-resolution confocal images of whole-mount skin preparations from CAR cKO and littermate F/F controls (ctrl) at E14.5 and E15.5 stained by immunofluorescence for LYVE-1 (green). Abnormal lymphatic vessels are seen in cKO embryos but not in controls at both time points. Scale bar = 50 µm. (TIFF) [file pone.0037523.s003.tif]

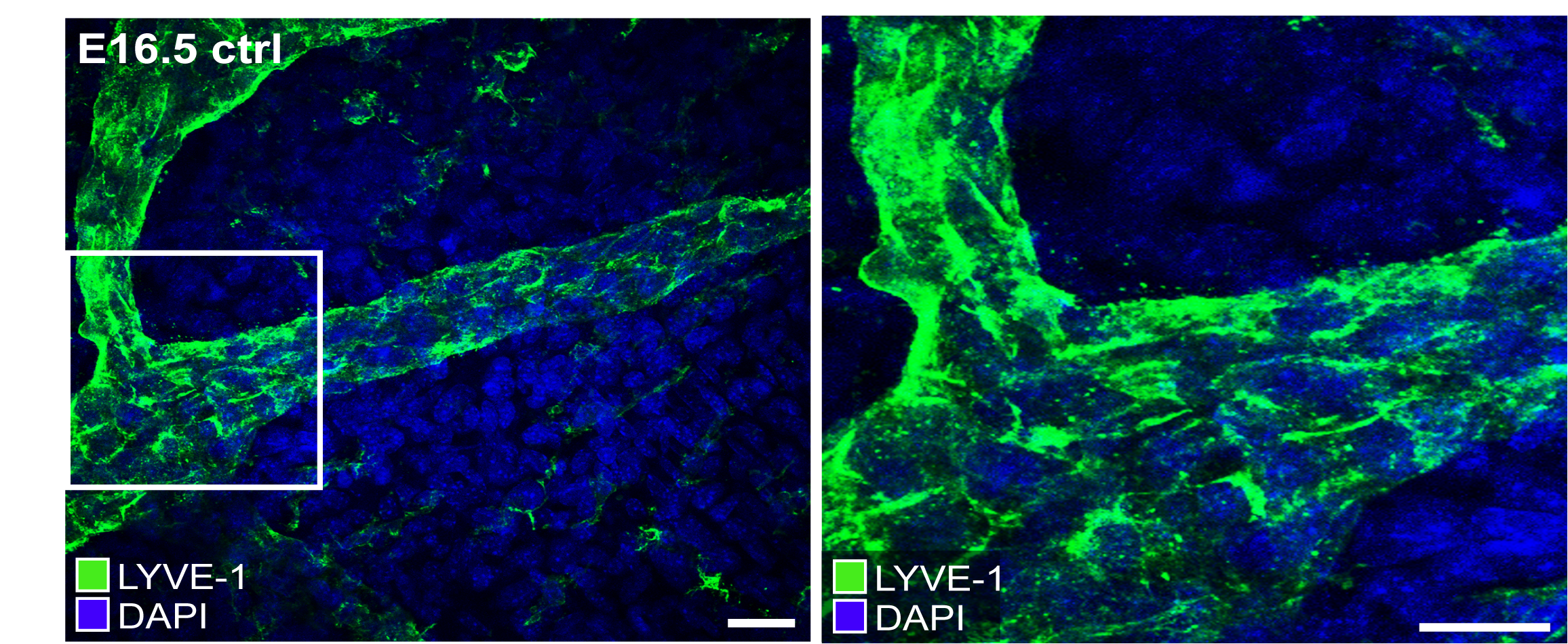

Supplement: Figure S4 — Lymphatic vessels of E16.5 ctrl embryos have no endothelial gaps. High-resolution confocal images of whole-mount skin preparations from E16.5 F/F control embryos stained by immunofluorescence for LYVE-1 (green). DAPI was used to visualize nuclei (blue). No endothelial gaps were present in lymphatic vessels of ctrl embryos. Scale bar = 150 µm. (TIFF) [file pone.0037523.s004.tif]
